# Unveiling the Excited State Dynamics of Indole in Solution

**DOI:** 10.1021/acs.jctc.3c00221

**Published:** 2023-06-17

**Authors:** Cheng Giuseppe Chen, Mauro Giustini, Marco D’Abramo, Andrea Amadei

**Affiliations:** † Department of Chemistry, Sapienza University of Rome, Rome 00185, Italy; ‡ Department of Technological and Chemical Sciences, Tor Vergata University of Rome, Rome 00133, Italy

## Abstract

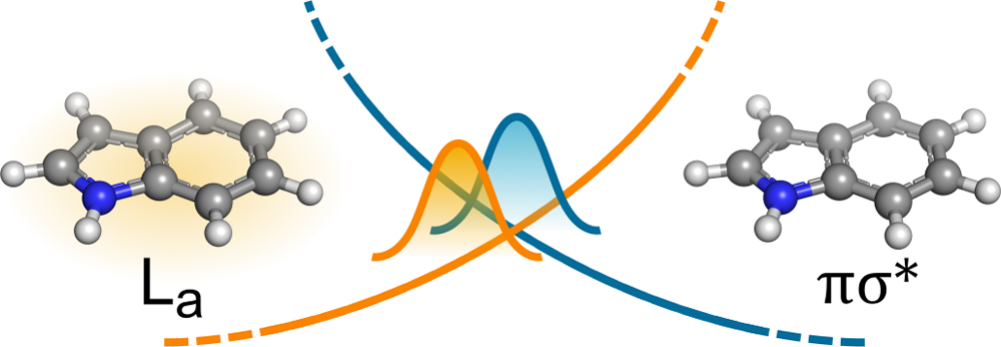

In this paper, we reconstruct in detail the dynamics of the emitting electronic excited state of aqueous indole, investigating its relaxation mechanism and kinetics to be related to the time-dependent fluorescence signal. Taking advantage of the results shown in a very recent paper, we were able to model the relaxation process in solution in terms of the transitions between two gas-phase singlet electronic states (^1^L_a_ and ^1^L_b_), subsequently irreversibly relaxing to the gas-phase singlet dark state (^1^πσ*). A comparison of the results with the available experimental data shows that the relaxation mechanism we obtain by our theoretical-computational model is reliable, reproducing rather accurately all the experimental observables.

## Introduction

**Figure 1:**
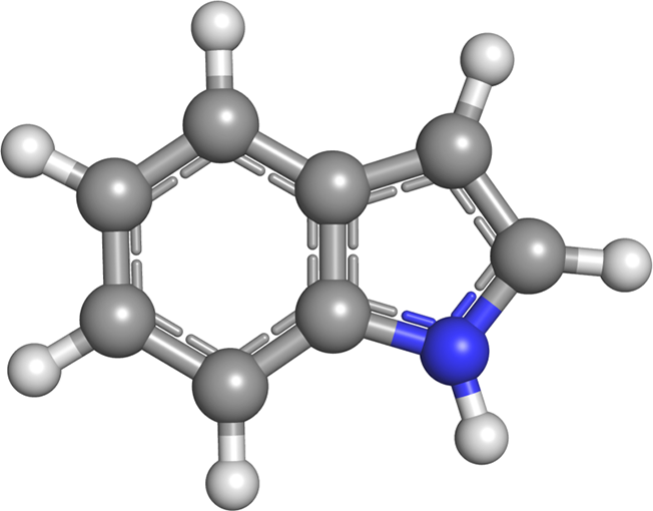
Graphical representation of the indole molecule. N in blue, C in gray, and H in white.

Indole (see Figure 1) represents one of the most studied model molecules for the absorption and emission properties of one of the few fluorescent amino acids found in natural proteins, i.e., tryptophan. Depending on the solvent, in the UV region, indole shows an absorption band lying in a relatively narrow region around 290 nm. Such an absorption band mostly arises from a pair of overlapping π → π* electronic transitions, conventionally labeled as ^1^L_a_ and ^1^L_b_ states^1^ (hereafter referred to as simply L_a_ and L_b_), nearly degenerate in energy (in ethanol, the 0–0 transitions occur at 290 and 294 nm, respectively^2^). Upon excitation, both geometrical and solvent relaxation occurs, thus resulting in the lowering of the energy of L_a_ and L_b_ states, in some cases leading even to their inversion in a polar environment, because of the higher dipole moment of the L_a_ state with respect to L_b_.^3^ This is at the basis of the complexity in the interpretation of the radiative deactivation processes of the electronic excited states that are responsible for the large, solvent-polarity dependent Stoke’s shift shown by indole (and, consequently, by tryptophan) and its high quantum yield. These properties have been exploited to probe local changes of polarity in proteins containing this amino acid upon denaturation^4,5^ or ligand binding,^6,7^ even though proteins, in general, contain more than one tryptophan residue, making it often extremely difficult to unambiguously assign the changes in fluorescence to a particular protein domain/site. This has led to the proposal in the literature of several analogues of this important amino acid, variously substituted on the indole moiety, in order to increase the sensitivity of this spectroscopic method in probing the protein structure and dynamics.^8^

Even though indole photophysics has been deeply investigated by several authors, both from an experimental^9−12^ and theoretical point of view,^13−16^ some unclear aspects still remain. For example, the exact mechanism of the excited electronic state relaxation (energy transfer) in polar solvents, such as water is still a matter of debate.

This paper will exploit the general model for treating electronic state transitions, focused specifically on charge transfer and intersystem crossing reactions, developed by our group in the past,^17,18^ to address the above-mentioned relaxation of the excited electronic states of aqueous indole, focusing on the thermodynamics and kinetics of the deactivation processes at the basis of the fluorescence properties of this model molecule. Such an approach has provided accurate results at significantly less computational expense when compared to the more commonly used strategies relying on the calculation of an ensemble of QM/MM trajectories.^19−21^ In fact, the high cost of this latter approach usually limits its applicability to the picosecond time scale, making it unsuitable for the current study. Even though Machine Learning-based approaches enable longer simulations,^22−24^ our method offers a much reduced overall computational cost permitting an extended phase space sampling, an easier implementation, and a robust and coherent quantum treatment, making it an appealing alternative, especially when the other approaches are not feasible.

## Theory

In a previous paper,^25^ we studied in detail both the absorption and emission vibronic spectra of aqueous indole (the quantum center, QC), as reconstructed by means of a theoretical–computational model based on the Perturbed Matrix Method (PMM) approach^26−28^ and MD simulations, providing at each MD frame the QC electronic Hamiltonian matrix *H̃*_e_ representing the electronic Hamiltonian operator *Ĥ*_e_ within the unperturbed electronic eigenstate basis set Φ_*j*_^0^ (i.e., the eigenstates of the noninteracting QC) via^28^
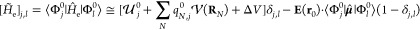
1where  is the energy of the *j*th unperturbed electronic eigenstate (i.e., the *j*th unperturbed electronic energy), *N* runs over the QC atoms with positions **R**_*N*_, *q*_*N*,*j*_^0^ is the *N*th atomic charge in the *j* unperturbed eigenstate,  is the perturbing electric potential at the *N*th atom position, the scalar function Δ*V* (independent of the electronic states) approximates all the higher order terms of the diagonal elements, **E**(**r**_0_) is the perturbing electric field at the reference position **r**_0_ (typically the QC center of mass), **μ̂** is the dipole operator, and δ_*j*,*l*_ is the Kronecker delta. By means of the eigenvectors and eigenvalues of the Hamiltonian matrix provided by eq 1, we can obtain any perturbed electronic property at each MD frame.

One of the main results of our previous paper^25^ was that the first perturbed electronic excited state of aqueous indole can be conceived as fluctuating among the first three gas-phase (i.e., unperturbed) singlet electronic excited states: the spectroscopically active L_b_ and L_a_ states and the dark state πσ*. The perturbed first excited electronic state is largely corresponding to the L_b_ electronic state just after the excitation (i.e., within the ground state ensemble), subsequently relaxing to a population mixture largely characterized by the L_a_ electronic state, thus providing an emission spectrum essentially corresponding to the L_a_ ensemble one. In this paper, we characterize in detail the transitions and processes occurring within the population of the perturbed electronic first excited state, reconstructing its relaxation kinetics and inherent reaction scheme in terms of the emission decay and of the interconversion rates among L_b_, L_a_, and πσ* (i.e., the diabatic states to be used for modeling the nonradiative relaxation). It is worth noting that the L_b_, πσ* and L_a_ electronic states correspond to the gas phase (i.e., unperturbed) first three electronic excited states, respectively, at the ground state optimized geometry where each of such excited states is characterized by well distinguishable diagonal and transition dipoles. We then defined the L_b_, πσ*, or L_a_ electronic state at each QC nuclear configuration via one of the unperturbed first three electronic excited states: the one best corresponding to the ground state optimized geometry L_b_, πσ*, or L_a_ state (e.g., comparing the diagonal and transition dipoles), regardless of its position within the energy order. When considering the unperturbed electronic excited state optimized geometries, providing the equilibrium structure/structures for each excited state, we obtained that while the L_b_ state is always corresponding to the first unperturbed electronic excited state, the πσ*, and L_a_ states exchange their energy position: i.e., the unperturbed second electronic excited state has two energy minima where it corresponds to either the L_a_ or the πσ* state. Note, however, that all such unperturbed excited state minima correspond to three different energy minima for the aqueous indole electronic first excited state (the perturbed electronic first excited state): i.e., in each minimum well (conformational basin) the perturbed first electronic excited state corresponds to either the L_b_, πσ*, or L_a_ state, respectively.^25^

In previous papers,^17,18^ we derived in detail a general model for treating diabatic state transitions, focusing specifically on charge transfer reactions^17^ modeled via using the unperturbed electronic states as the proper diabatic states. In this paper, we apply the same theoretical–computational model using the L_b_, πσ*, and L_a_ unperturbed electronic states as the appropriate diabatic states to reconstruct the kinetics of the perturbed electronic first excited state relaxation (energy transfer) in aqueous indole.

### Modeling Absorption and Emission Spectra

We reconstructed the aqueous indole emission properties by means of the procedure described in detail in our previous paper^25^ where we modeled the absorption and fluorescence spectra due to the singlet vibronic state transitions (i.e., we disregard any singlet → triplet excitation/emission). Briefly, for each electronic excitation/emission of the chromophore, i.e., the quantum center (QC), by extracting the MD frames of the ground/excited state simulation with the *i*th perturbed electronic excited eigenstate best corresponding to a given *j*th unperturbed one and for a flexible QC also belonging to a single conformation (MD subensemble), we obtain the corresponding excitation or emission vertical electronic spectrum by means of the electronic vertical energy *h*ν (with *h* the Planck constant and ν the vertical electronic transition frequency) and transition dipole **μ**_0,*i*_ as provided by the Perturbed Matrix Method^28^ (PMM), via^25,29^
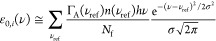
2
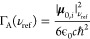
3
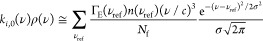
4
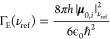
5with ϵ_0_ being the vacuum dielectric constant, *ℏ* = *h*/2π the summation running over the frequency bins (i.e., the tiny grid intervals discretizing the spectral frequency range) identified by the vertical electronic transition reference frequencies ν_ref_, *n*(ν_ref_) the number of (extracted) MD frames with the vertical electronic transition frequency within the bin centered in ν_ref_, |**μ**_0,*i*_|_ν_ref__^2^ the corresponding electronic vertical transition dipole mean square norm as obtained averaging over the bin MD frames, *N*_f_ the total number of extracted MD frames, and the Gaussian distributions providing the broadening due to the QC semiclassical vibrations disregarded by the PMM calculations (i.e., the classical-like vibrational motions due to the low frequency modes). Note that with conformation we mean a configurational region of the QC internal semiclassical coordinate space (typically corresponding to a harmonic or quasi-harmonic basin) where all the QC electronic vertical transition properties, except the vertical transition energy, can be obtained at a single reference structure corresponding to a local energy minimum of the unperturbed ground state (excitation) or of the *j*th unperturbed electronic excited state best corresponding to the perturbed *i*th excited state (emission). On the basis of the assumed negligible perturbation effects on the vibrational eigenstates and eigenvalues, we can reconstruct for each subensemble the *m*th vibronic spectral peak by multiplying the absorption and emission vertical electronic spectrum by |⟨φ_0,0_^0^|φ_*j*,*m*_^0^⟩|^2^ and |⟨φ_*j*,0_^0^|φ_0,*m*_^0^⟩|^2^, respectively, with φ_0,0_^0^, φ_0,*m*_^0^ being the ground and *m*th vibrational states of the unperturbed ground electronic state and φ_*j*,*m*_^0^, φ_*j*,0_^0^ the *m*th and ground vibrational states of the unperturbed *j*th electronic excited state (the unperturbed electronic state best corresponding to the *i*th perturbed electronic excited state within the subensemble), as well as by substituting in eqs 2 and 4 the vertical electronic transition reference frequencies ν_ref_ with the bin reference frequencies of the *m*th vibronic transition ν_ref_^*m*^, given by^25^
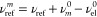
6where ν_*m*_^0^ is the frequency of the unperturbed *m*th vibronic transition (unperturbed *m*th vibronic frequency) possibly corrected to match the experimental gas-phase value, ν_el_^0^ is the frequency of the unperturbed vertical electronic transition (unperturbed electronic vertical frequency), and clearly *n*(ν_ref_^*m*^) = *n*(ν_ref_). Note that we evaluate the vibrational state overlaps via the simplified very efficient procedure described in the previous paper^25^ based on considering identical vibrational modes and frequencies for the ground and excited electronic states, with the vibrational wave functions of corresponding modes only differing for their minimum energy positions. The sum of these vibronic peaks provides the subensemble vibronic spectrum, and thus summing such spectra over all the subensembles, each statistically weighted by the corresponding probability as provided by the ground/excited state MD simulation, and then over all the relevant electronic transitions we obtain the complete vibronic spectrum.

### Modeling the Singlet Electronic Excited State Transitions

**Figure 2:**
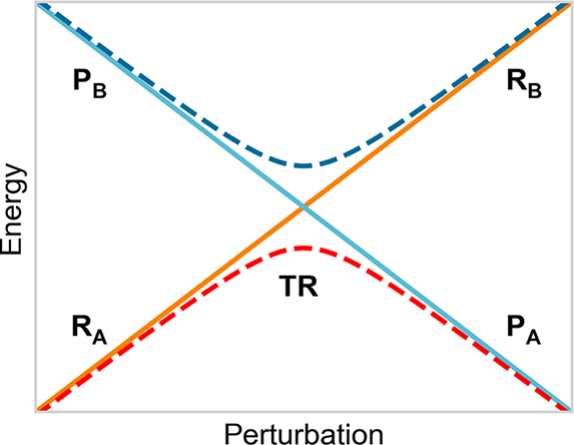
Schematic description of the reactant R and product P diabatic energy surfaces (solid lines) as well as of the related lower (A) and higher (B) adiabatic energy surfaces (dashed lines) for a single diabatic energy crossing.

**Figure 3:**
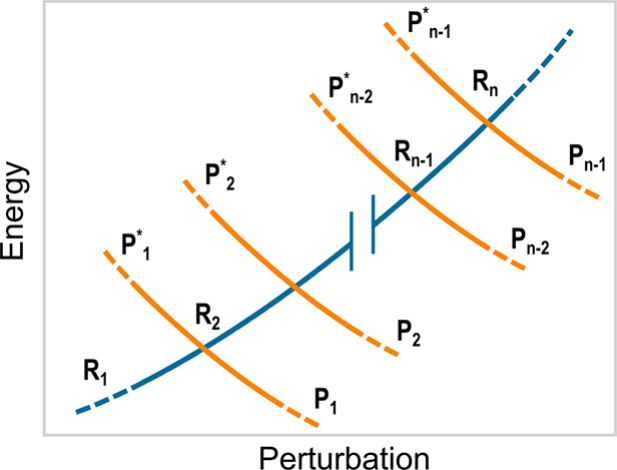
Schematic picture of the reaction process possibly involving several vibronic state crossings (reaction steps) defined by the R diabatic vibronic state and the relevant P diabatic vibronic states (i.e., the P vibronic states involved in each reaction event).

Defining for a rigid/semirigid QC, or for a conformational basin of a flexible QC, with A and B the two adiabatic energy surfaces (see Figure 2) involved in the electronic state transition defined by the reactant (R) and product (P) diabatic states (the L_b_, L_a_, and πσ* unperturbed electronic states), the rate equations for the R → P reaction can be obtained when considering all the reaction steps due to the crossing of the R vibronic energy surface with all the P vibronic ones involved in each reaction event (see Figure 3). Note that each diabatic vibronic energy surface is defined by the vibronic energy of the diabatic state considered as a function of the perturbation acting on the QC, i.e., as a function of the classical-like degrees of freedom determining the perturbing field due to the atomic-molecular environment and (if present) the QC semiclassical deformations, both parametrically defining the vibronic wave functions and thus determining the vibronic energies. Assuming for each reactive trajectory the *R*_1_ → *R*_*n*_ reaction steps as completely irreversible with stationary intermediates (irreversible reaction step approximation^17^), *R*_A_ corresponding to the *R*_1_ substate of Figure 3 and being the initial reactant condition, we obtain^17^
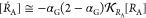
7
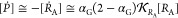
8where α_G_ is the reaction transmission coefficient,^17^ as obtained by the transition region (TR) crossings via the Landau–Zener approximation, and  is the reaction kinetic constant providing
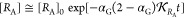
9
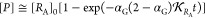
10with [*R*_A_]_0_ being the *R*_A_ concentration at the beginning of the reaction. In the following, we will always consider the electronic state transition as occurring according to the previous equations: i.e., *R*_A_ is the initial reactant state and hence for each reactive trajectory the transition energy (i.e., the diabatic energy difference) is always positive before the first crossing is achieved. It is worth noting that the *R* and *P* diabatic electronic energies, furnishing via their difference the relevant transition energy of the process and providing when vanishing the first crossing of the *R*_A_ trajectories, are provided by the corresponding (perturbed) electronic Hamiltonian matrix diagonal elements (i.e., [*H̃*_e_]_*R*,*R*_ and [*H̃*_e_]_*P*,*P*_) each as obtained at the proper unperturbed electronic state minimum energy geometry^17^ (note that we always assume identical *R* and *P* zero point vibrational energies).

Equations 7–10 provide the link between the chemical kinetics model and the data obtained by the computational simulation. In fact, by means of a large set of proper MD simulations in combination with quantum calculations it is possible, for a given *R* and *P* diabatic state couple, to evaluate the distribution of the time-lengths needed by the QC for reaching the first diabatic energy surface crossing in the reactant ensemble and thus to reconstruct the kinetic trace of [*R*_A_] providing the corresponding kinetic constant , as shown in previous papers.^17^ Moreover, by means of the Landau–Zener approximation, we can estimate the reaction transmission coefficient for the subset of reactive trajectories unidirectionally (i.e., irreversibly) traversing a given identical number *n*_P_ of *P* (subsequent) vibronic states and characterized by a virtually identical electronic adiabatic fraction χ_e_ that we consider roughly constant over the reaction steps, via^17^
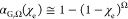
11
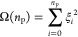
12In the last equations, χ_e_ corresponds to the electronic adiabatic fraction as obtained at the first crossing encountered by the reactive trajectories and ξ_*i*_^2^ = |⟨φ_*R*,0_^0^|φ_*P*,*i*_^0^⟩|^2^ is the squared (unperturbed) vibrational state overlap of the *R* (ground) and *P* (*i*th) vibrational states (see Figure 3), as obtained by assuming for the *R* and *P* diabatic states identical vibrational modes and frequencies with wave functions of corresponding modes differing only for their minimum energy positions.^17,18^ Therefore, when considering the first crossing for all the reactive trajectories (providing different values of χ_e_), we obtain by averaging over all the crossing trajectories
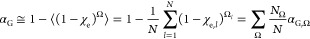
13
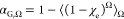
14where *∑*_Ω_*N*_Ω_ = *N* is the total number of crossing trajectories (each with χ_e,*l*_ electronic adiabatic fraction and Ω_*l*_ squared vibrational state overlap sum), the angle brackets mean as usual averaging over all the crossing trajectories, and the angle brackets with subscript Ω indicate averaging over the *N*_Ω_ crossing trajectories irreversibly traversing a given identical number of *P* (subsequent) vibronic states. Note that when dealing with typical charge transfer processes each reaction event may involve the irreversible traversing of roughly the complete set of the productive *P* vibronic diabatic energy surfaces (i.e., corresponding to non-negligible ξ_*i*_^2^), thus providing for all the crossing trajectories Ω ≈ 1 (i.e., α_G_ ≈ α_e_). Differently, for electronic state transitions with reaction events involving the irreversible traversing of only a subset of the productive *P* vibronic diabatic energy surfaces, we necessarily have ξ_0_^2^ ≤ Ω < 1 with ξ_0_^2^ the squared vibrational state overlap of the first relevant *R*, *P* vibronic crossing encountered by the *R* ensemble trajectories (i.e., for the *R*_A_ ensemble the vibronic crossing corresponding to the *P* vibrational ground state). In such more general cases, either we use eq 13 (requiring evaluation for each crossing trajectory the corresponding χ_e,*l*_ and Ω_*l*_) or we can employ the approximation
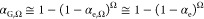
15providing
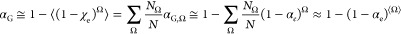
16
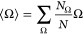
17where α_e,Ω_ = ⟨χ_e_⟩_Ω_ is the electronic transmission coefficient as obtained by evaluating the electronic adiabatic fraction χ_e_ at each of the *N*_Ω_ first crossings and then averaging over all these crossings and α_e_ = ⟨χ_e_⟩ is the electronic transmission coefficient as obtained averaging over all the *N* first crossings sampled by the MD simulations. Finally, the electronic adiabatic fraction χ_e_, as provided by the Landau–Zener and the dipolar approximations, can be obtained via^17^
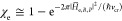
18
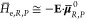
19with **E** being the perturbing electric field at each (first) crossing as obtained at the QC mass center and *H̅*_e,R,P_ and **μ̅**_R,P_^0^ being the arithmetic mean of the *R*, *P* electronic couplings and electronic reactant–product unperturbed transition dipoles as obtained at the *R* and *P* energy minima.

In the unfortunate case that the kinetics is too slow to obtain a proper crossing sampling from MD simulations (as for some of the electronic state transitions investigated in this paper), we can still reasonably well evaluate  assuming an approximately Gaussian behavior for the diabatic energy difference (the transition energy ) fluctuations around the transition energy mode value (i.e., the transition energy corresponding to the probability distribution maximum), , with such a Gaussian range including the diabatic energy crossing, i.e.,  defining the first crossing encountered by the *R*_A_ trajectories (note that the *R*_A_ subscript of the angle brackets indicates that averaging is performed in the *R*_A_ equilibrium ensemble). In fact, considering that within each TR we have a virtually unidirectional flux, we obtain^18^
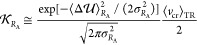
20where σ_*R*_A__^2^ is the variance of the transition energy within the *R*_A_ equilibrium ensemble and ⟨*v*_cr_⟩_TR_ is the equilibrium average of the crossing velocity norm *v*_cr_, which can be evaluated by averaging the norm of the transition energy time derivative *v* within the *R*_A_ equilibrium ensemble (i.e., ⟨*v*_cr_⟩_TR_ ≅ ⟨*v*⟩_*R*_A__).

Similarly, when no crossing sampling is present we can also estimate the transmission coefficient according to eq 16, i.e., α_G_ ≈ 1 – (1 – α_e_)^⟨Ω⟩^ (with ξ_0_^2^ ≤ ⟨Ω⟩ ≤ 1), by using the electronic transmission coefficient α_e_ = ⟨χ_e_⟩ as provided by the crossing mean coupling approximation based on expressing 1 – ⟨χ_e_⟩ = ⟨exp[–2π|*H̅*_e,*R*,*P*_|^2^/(ℏ*v*_cr_)]⟩ via
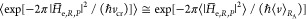
21
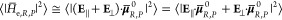
22
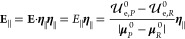
23
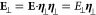
24
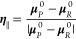
25
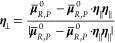
26where  and **μ**_*R*_^0^, **μ**_*P*_^0^ are the electronic energies and diagonal dipoles of the *R* and *P* diabatic (unperturbed) states (each as obtained at the corresponding unperturbed electronic state energy minimum), **E**_∥_ provides the perturbing field along the unit vector **η**_∥_ at each first crossing event, following from
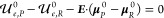
27approximating the crossing condition within the dipolar approximation,^17^**E**_⊥_ is the perturbing field along the unit vector **η**_⊥_ orthogonal to **η**_∥_ (note that in general **E** ≠ **E**_∥_ + **E**_⊥_), and according to eq 26, we have used
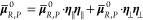
28clearly providing
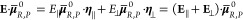
29

From eq 29, we readily obtain (considering that we deal with real electronic eigenstates and thus real electronic transition dipoles)
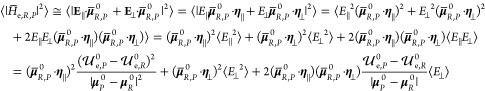
30where we used the fact that for a rigid/semirigid QC (for a flexible QC considering a single conformational basin) **μ̅**_R,P_^0^·**η**_∥_ and **μ̅**_R,P_^0^·**η**_⊥_, just like , are fixed values independent of the instantaneous crossing configuration. From the last equation, it follows that if *E*_∥_ and *E*_⊥_ can be considered as statistically independent variables (see Figures S1 and S2 in the SI), then we can assume that the distribution of *E*_⊥_ within the *R*_A_ equilibrium ensemble be virtually identical to the one obtained over all the crossing events where , and hence
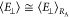
31
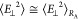
32readily providing
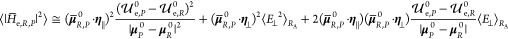
33

Equation 33 plainly shows that within the approximations used we can estimate the electronic transmission coefficient α_e_ using the equilibrium *R*_A_ ensemble, via ⟨*E*_⊥_⟩_*R*_A__, ⟨*E*_⊥_^2^⟩_R_A__, and ⟨*v*_cr_⟩_*R*_A__ once we have evaluated  and (**μ̅**_*R*,*P*_^0^·**η**_∥_), (**μ̅**_*R*,*P*_^0^·**η**_⊥_).

## Computational Details

The reference QM geometries used for the PMM calculations were obtained by optimizing the structure of indole in the electronic ground state and L_b_, L_a_, and πσ* (unperturbed) excited states using CCSD and EOM-CCSD, and 6-311+G(d) as the basis set. The optimization of the unperturbed electronic ground state and of the L_b_ and L_a_ electronic states was performed in vacuo, whereas the minimized geometry of the dark πσ* state was estimated in aqueous solution (modeled using IEFPCM^30^): i.e., we assume for these unperturbed electronic states identical minimum energy structures, at least for the quantum nuclear coordinates, for the isolated or the perturbed QC, with a virtually identical perturbed and unperturbed dark state at the corresponding minimum energy geometry. Note that for the dark state, we were not able to reach convergence of the calculation in vacuo, possibly due to the very shallow minimum of this unperturbed electronic state in the isolated indole.^31^ The excited state minima were obtained by using the standard procedure of the Gaussian package,^32^ starting the calculation from the ground state optimized geometry where the first three gas-phase (unperturbed) electronic excited states correspond to the L_b_, πσ*, and L_a_ state, respectively. The procedure used by Gaussian minimizes over the electronic energy surfaces, conserving the character of the electronic state initially selected, by performing state-tracking procedures.^33^ Therefore, in our case we find a minimum for each of the three starting electronic states: the L_b_ minimum corresponding to the first unperturbed excited state minimum and the πσ* and L_a_ state minima corresponding to two different minima of the second unperturbed excited state. However, all such minima correspond to three different minima of the electronic perturbed first excited state: i.e., in the L_b_, πσ*, and L_a_ conformational basins (corresponding to the three optimized geometries) the perturbed first excited state is basically identical to the L_b_, πσ*, and L_a_ electronic state, respectively. For each of the optimized geometries (reference geometries), the electronic properties of the unperturbed ground and the lowest-lying six singlet excited states were calculated using CCSD and EOM-CCSD, and using 6-311+G(d) as the basis set. Like in our previous work,^25^ the calculated gas phase vibronic transition energies involving the L_b_ and L_a_ states were corrected to match the experimental gas phase 0–0 transitions reported in the literature:^34^ the shifts of −0.32 eV for GS → L_b_ and −0.57 eV for GS → L_a_ were used to correct the corresponding spectral signals and furnished the energy corrections for the L_b_*⇌* L_a_ reaction rate calculations. Since such gas-phase experimental data is not achievable for the πσ* (dark) state, no corrections have been applied when considering the transition energies involving the dark state (i.e., in the reaction rate calculations for L_b_ ⇌ πσ* and L_a_ ⇌ πσ*).

Furthermore, the vibrational eigenstates were calculated for each reference geometry using TD-DFT with M06-2X^35^ functional and 6-311+G(d) basis set. The corresponding vibronic transitions were calculated using the simplified treatment we proposed in a recent paper.^25^ In fact, the high computational cost of the more accurate QM methods did not allow us to use EOM-CCSD for both the optimization and calculation of the vibrational frequencies, a condition required with the commonly used algorithms.^36,37^ In our approach, the use of different levels of theory was rigorously justified by separating and uncoupling the vibrational modes between classical-like (i.e., those modes with energy gaps less than *k*_B_*T*) and the quantum modes (i.e., all the other modes). Vibrational modes belonging to different electronic states with similar frequencies were approximated as being virtually identical, differing only in the positions of their minimum energy, an assumption that is further validated by the quasi-diagonal Duschinsky matrices obtained for the considered electronic transitions (see Figure S3 in the SI). Moreover, the efficiency of our treatment allowed us to explicitly consider more than a million vibronic transitions without necessarily requiring the calculations being performed in a local minimum, as is mandatory by the commonly used method implemented in Gaussian.^32,36^ Note that the harmonic approximation for the vibrational states at each reference geometry is applicable even when dealing with a shallow minimum. In fact, by separating classical-like and quantum vibrational coordinates as allowed by our treatment, we can express the vibronic states at each reference geometry via only the quantum vibrational subspace defined by the vibrational modes with excitation energy higher than the thermal energy (i.e., those modes which are reasonably stiff to permit the quantum harmonic approximation), with all the lower frequency modes treated as classical-like (possibly anharmonic) coordinates and determining parametrically the vibronic wave functions. Therefore, in order to ensure an accurate harmonic approximation in modeling the vibrational wave functions involved in the vibronic states, we only have to assume no mixing of the classical-like and quantum nuclear coordinate subspaces with no significant coupling between them. In our previous paper,^25^ such an approach has already been applied to calculate both the absorption and emission vibronic spectra of indole providing accurate results when compared to the experimental data, therefore fully justifying its application in the present work. The benchmarking which led to the choice of functionals and basis sets is described in our previous work.^25^ The geometry optimizations, the calculation of the vibrational eigenstates and of the ESP charges^38,39^ of all the unperturbed electronic states were performed using Gaussian 16,^32^ while all the other electronic properties were calculated using Q-Chem 5.3.^40^

NVT MD simulations were performed for indole and 1276 TIP3P water molecules in a cubic box with sides of 3.37589 nm using periodic boundary conditions, at 300 K, for the ground state and the L_b_, L_a_, and πσ* excited states. Each excited state MD simulation was performed using the atomic charges of the diabatic excited state corresponding at each reference geometry to the perturbed first electronic excited state, with all the other force field parameters identical to those of the ground state force field (i.e., the GS optimized geometry is used as the excited state force field equilibrium structure). This choice is motivated by the tiny structural changes among the excited state minima and between these and the ground state (GS) one, providing for indole virtually indistinguishable geometries in the MD simulations (we tested the accuracy of this approximation for the L_b_ state by using the L_b_ reference geometry instead of the GS one as the excited state force field equilibrium structure, obtaining indistinguishable results). Note however that all the PMM calculations and reaction rate evaluations are obtained by using at each MD frame the proper excited state reference geometry with the corresponding unperturbed quantum states: at each MD frame the proper QC excited state reference structure is (mass-weighted) fitted to the instantaneous QC MD configuration to obtain the correct calculations involving the perturbing field.

**Figure 4:**
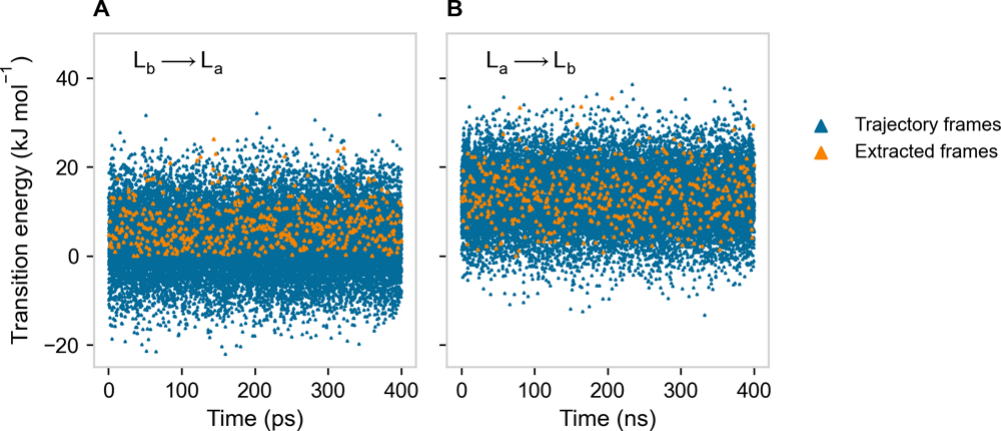
Instantaneous transition energies (blue) as obtained from the equilibrium MD simulations in the L_b_ ensemble, L_b_ → L_a_ transition (A), and in the L_a_ ensemble, L_a_ → L_b_ transition (B). The MD frames which were taken as the starting points for the nonequilibrium MD simulations used to reconstruct the kinetics of the transitions via the explicit crossing sampling are highlighted in orange.

The volume of the box was chosen in order to reproduce the experimental condition of isobaric insertion of the solute in liquid water at infinite dilution.^41^ The force field used was CHARMM36,^42,43^ and for the simulations of indole in the diabatic excited states (i.e., L_b_, L_a_, and πσ*) the atomic charges were replaced with the ESP charges obtained using EOM-CCSD/6-311+G(d) (see Figure S4 in the SI). The canonical sampling was achieved using the velocity rescaling thermostat.^44^ Each equilibrium simulation used to obtain the *R*_A_ equilibrium ensemble was carried out for 400 ns with a time step of 2 fs, using for the MD-PMM calculations 20,000 frames taken every 20 ps. Additional MD simulations, 500 trajectories of 3 ps for the L_b_ ensemble and 500 trajectories of 10 ps for the L_a_ ensemble, with 1 fs of time step were performed for obtaining the nonequilibrium simulations providing the explicit crossing sampling. The starting configurations of the nonequilibrium simulations were extracted from their corresponding equilibrium simulation by dividing the latter into 500 subtrajectories of 40 frames each and by randomly selecting a frame. For each subtrajectory, frames were randomly chosen and discarded until the resulting transition energy of the considered process (e.g., L_b_ → L_a_ in the L_b_ ensemble) was positive (see Figure 4), in which case such configuration was stored. Moreover, we performed for each diabatic state ensemble a 50 ps MD trajectory (with 1 fs time step) to obtain the corresponding equilibrium average crossing velocity norm to be used within the Gaussian and crossing mean coupling approximations. All the MD simulations were performed using Gromacs 2020.1,^45^ while the PMM calculations furnishing both the relaxation reaction properties and the emission spectra were performed using the open source program PyMM.^46^

## Results and Discussion

**Figure 5:**
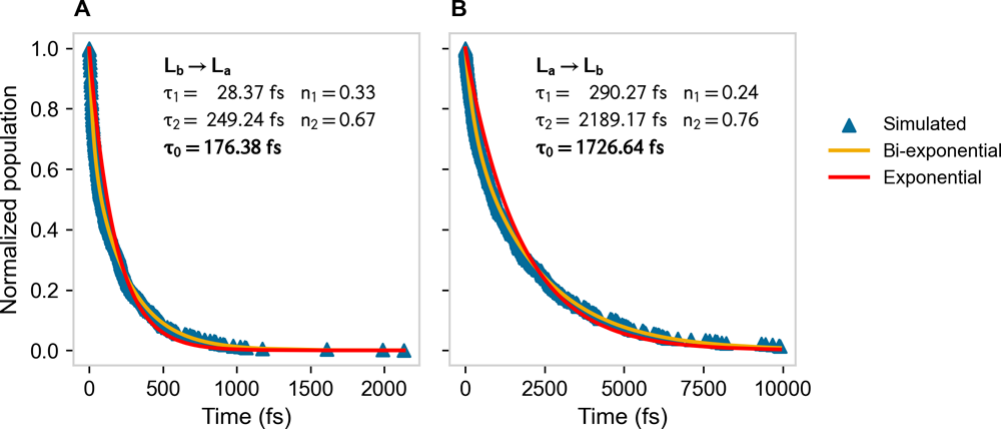
Kinetic traces (blue triangles) associated with the fully adiabatic L_b_ → L_a_ (A) and L_a_ → L_b_ (B) transitions, as obtained by monitoring the corresponding diabatic energy crossings provided by 500 different MD trajectories. The fitted biexponential curves n_1_ e^–*t*/τ_1_^ + n_2_ e^–*t*/τ_2_^ with n_1_ + n_2_ = 1 (yellow lines) are shown, as well as the exponential curves e^–*t*/τ_0_^ (red lines) each resulting from the mean lifetime τ_0_ as obtained by the weighted average of the corresponding fitted biexponential curve mean lifetimes (i.e., τ_0_ = n_1_τ_1_ + n_2_τ_2_). Note that in our model we assume a time-independent distribution within the *R*_A_ ensemble (i.e., ensuring a single *R*_A_ kinetic state) and thus the slight inaccuracy of the exponential curves (possibly due to *R*_A_ inner relaxations kinetically comparable to the L_b_*⇌* L_a_ reaction) is disregarded and then the overall mean lifetime τ_0_ is used to obtain the reactant fully adiabatic kinetic constant .

From previous results,^25^ we know that just after the excitation process the first (perturbed) electronic excited state is largely coinciding with the L_b_ electronic diabatic state, with the excited state population subsequently relaxing via transitions to the L_a_ and the dark (πσ*) electronic diabatic states. According to the theoretical model described in the theory section, we estimated all the relevant kinetic properties for each of the diabatic state transitions (the transitions among L_b_, L_a_, and πσ*) by means of MD simulations performed in the corresponding reactant ensemble (note that for each of such ensembles, the perturbed first electronic excited state is basically coinciding with the corresponding diabatic state, ensuring fully coherent results). By using the L_a_, L_b_, and πσ* MD ensembles, we then obtained the kinetic properties for all the possible transitions, as summarized in Table 1. Due to the slow kinetics of the transitions from/to the dark state, we were able to obtain a proper crossing sampling and the corresponding explicit kinetics, as provided by 500 independent nonequilibrium trajectories, only for the L_b_*⇌* L_a_ interconversion (see Figures 4 and 5). For the other transitions involving the dark state, we used the Gaussian and crossing mean coupling approximations (see the theory section) for evaluating the corresponding kinetics.

**Table 1:** Mean Transition Energy , Standard Deviation of the Transition Energy σ_*R*_A__, Mean Crossing Velocity Norm as Provided by the *R*_A_ Mean Transition Energy Time Derivative Norm ⟨*v*⟩_*R*_A__, Fully Adiabatic Transition Mean Lifetime , Transmission Coefficient α_G_, and Reaction Mean Lifetime τ for the Relevant Diabatic State Transitions of the Perturbed Electronic First Excited State Population^a^

transition	(kJ mol^–1^)	σ_*R*_A__ (kJ mol–1)	⟨*v*⟩_*R*_A__ (kJ mol^–1^ fs^–1^)	τ_0_	α_G_	τ
L_b_ → L_a_	3.62 ± 0.05	7.1	0.4	105 ± 1 fs	0.859 (1)	107 (105) fs
				176 ± 8 fs^b^	0.295^b^	350 ± 23 fs^b^
L_a_ → L_b_	13.15 ± 0.05	6.5	0.4	614 ± 11 fs	0.854 (1)	627 (614) fs
				1727 ± 118 fs^b^	0.422^b^	2593 ± 225 fs^b^
L_b_ → πσ*	127.36 ± 0.26	31	1.7	461 ± 21 ps	0.021	11.0 ± 0.5 ns
L_a_ → πσ*	138.00 ± 0.25	33	2.0	458 ± 46 ps	0.065	3.7 ± 0.4 ns
πσ* → L_b_	213.58 ± 0.39	37	2.4	1676 ± 347 ns	0.032	26.30 ± 5.44 μs
πσ* → L_a_	270.69 ± 0.47	40	2.6	475 ± 130 μs	0.114	2.20 ± 0.60 ms

a The noise shown corresponds to a standard error while when not reported the noise magnitude is equal to or lower than the magnitude of the referred property last digit. The mean lifetimes and transmission coefficient were calculated using the Gaussian approximation and the crossing mean coupling approximation, respectively, except when specified by the footnote. Note that  is always defined by the transition scheme indicated in the table, , and in the transitions from/to the πσ* diabatic state, we always evaluated α_G_ ≈ 1 – (1 – α_e_)^⟨Ω⟩^ and the related τ by using ⟨Ω⟩ = ξ_0_^2^. Finally, for the L_a_, L_b_ interconversion, we provide α_G_ ≈ 1 – (1 – α_e_)^⟨Ω⟩^ and related τ values (Gaussian and crossing mean coupling approximations) as obtained via using either ⟨Ω⟩ = ξ_0_^2^ or ⟨Ω⟩ = 1 (the latter shown between parentheses), while when using the simulation crossing sampling (indicated by the footnote), we provide the values of α_G_ ≅ 1 – ⟨(1 – χ_e_)^Ω^⟩ and related τ (see the theory section). For this latter approach, additional information on the estimated errors is provided in Figures S6 and S7 in the SI.

b Mean lifetime and transmission coefficient obtained via the explicit MD sampling of the diabatic energy crossings.

It is worth remarking that given the large  values involved in the transitions from/to the dark state (see Table 1), we always assume in such reactions that only the first crossing is accessible (i.e., the *R* energy region beyond the first crossing is too unstable for allowing further crossings before inverting the traversing velocity), and thus we obtained the corresponding transmission coefficients via α_G_ ≈ 1 – (1 – α_e_)^⟨Ω⟩^ with ⟨Ω⟩ = ξ_0_^2^.

For the L_a_, L_b_ interconversion, the values of α_G_ ≅ 1 – ⟨(1 – χ_e_)^Ω^⟩ and related τ were evaluated by the simulation crossing sampling (the distributions of the sampled Ω values are shown in Figure S5 in the SI). For comparison, the Gaussian and crossing mean coupling approximations with either ⟨Ω⟩ = ξ_0_^2^ or ⟨Ω⟩ = 1 were also used to estimate α_G_ ≈ 1 – (1 – α_e_)^⟨Ω⟩^ and τ values. It is worth noting that the L_b_*⇌* L_a_ reaction involves insufficiently large  values for accurately employing the Gaussian approximation: i.e., possible nonequilibrium *R*_A_ inner distribution due to *R*_A_ inner relaxations kinetically comparable to the L_b_*⇌* L_a_ reaction. However, the τ_0_ and α_G_ values obtained by means of the Gaussian and crossing mean coupling approximations (the latter when using ⟨Ω⟩ = ξ_0_^2^) are reasonably close to the corresponding values as provided by the simulation explicit crossing sampling, reproducing the correct order of magnitude of the reaction mean lifetimes and thus confirming the reliability of these approximations needed when no crossing sampling is achievable (for the L_b_ → πσ* and L_a_ → πσ* transitions where  is much larger we expect a higher accuracy of the Gaussian approximation).

**Figure 6:**
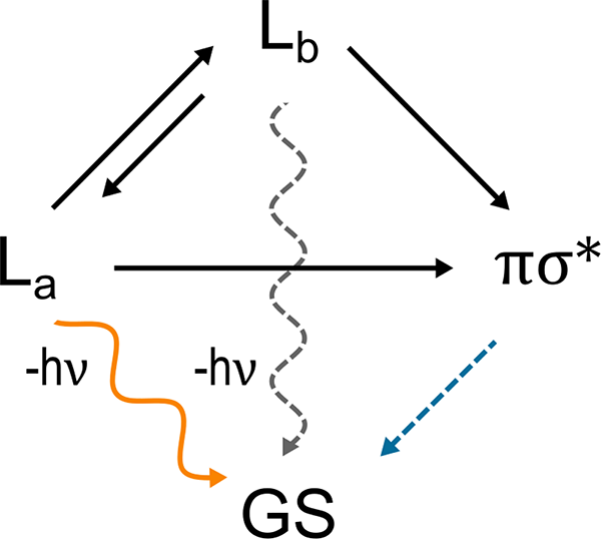
Schematic description of the relaxation mechanism of aqueous indole first excited state population, according to our theoretical–computational model.

**Figure 7:**
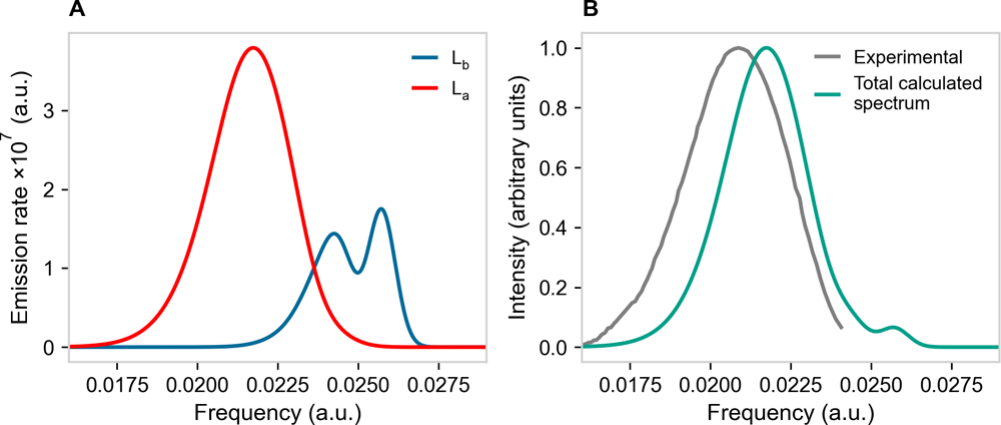
(A) Calculated vibronic emission spectra of the perturbed first electronic excited state, as obtained by the L_b_ (i.e., ) (blue) and L_a_ (i.e., ) (red) MD ensembles. (B) Calculated vibronic emission spectrum of the perturbed electronic first excited state (green) as obtained from the equilibrium weighted average of the L_b_ and L_a_ emissions (see Table 3) compared to the experimental vibronic emission spectrum (gray). The experimental emission spectrum of indole in aqueous solution is adapted from Hilaire et al.^52^

From Table 1, it is evident that the L_b_*⇌* L_a_ reaction is much faster than any transition toward the dark state (in line with the available computational and experimental data for aqueous tryptophan^21,47^), with the latter being a virtually irreversible transition which leads to the overall relaxation mechanism of aqueous indole first (perturbed) electronic excited state represented in Figure 6. Note that the dark to ground state (πσ* → GS) relaxation has not been investigated in this paper as the dark state radiative relaxation is negligible; that is, we can reconstruct the fluorescence data by considering only the L_b_*⇌* L_a_ interconversion, their radiative relaxations to the ground state, and the L_b_ → πσ* and L_a_ → πσ* (irreversible) transitions (the  and  radiative relaxations are fully negligible). Moreover, given the much faster kinetics of the L_b_*⇌* L_a_ reaction compared to their radiative relaxations to GS as well as compared to the L_b_ → πσ* and L_a_ → πσ* transitions, we can safely assume full equilibrium for the L_b_, L_a_ mixture during the relaxation process. Therefore, according to Figure 6 we can express the radiative τ_r_ and nonradiative τ_nr_ relaxation mean lifetimes of the first electronic excited state to be compared to the experimental ones, via
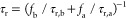
34
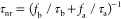
35with τ_r,b_, τ_r,a_ being the radiative relaxation mean lifetimes for the  and  emissions (see Figure 7 and Table 2), τ_b_, τ_a_ the reaction mean lifetimes of the L_b_ → πσ* and L_a_ → πσ* transitions (see Table 1), and *f*_a_, *f*_b_ the L_a_, L_b_ equilibrium fractions given by
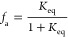
36
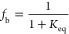
37
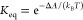
38where *K*_eq_ is the equilibrium constant for the L_b_*⇌* L_a_ reaction and Δ*A* is the corresponding free energy change defined by the L_b_ → L_a_ transition (see Table 3). Note that the reaction free energy Δ*A* is obtained by
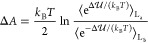
39where  is the L_b_ → L_a_ transition energy and the L_a_, L_b_ subscripts of the angle brackets mean averaging within the equilibrium L_a_ and L_b_ ensemble, respectively. Interestingly, from Figure 7 it appears that although the calculated L_b_ emission is by far less relevant, it is still able to provide a specific tiny peak at the higher energy edge of the spectrum, located beyond the experimentally accessible energy range. However, it is worth noting that such a residual L_b_ radiative effect might be due to the overestimated equilibrium L_b_ population, as suggested by the better matching with the experimental radiative relaxation mean lifetime as obtained when considering only the L_a_ population (see Table 2). Such slight discrepancies, possibly stemming from the different details between the computational model system we used and the experimental one, are to be expected as the physical–chemical behavior reconstructed by the theoretical–computational model cannot reproduce exactly all the experimental conditions even when capturing the essential physics of the process studied.

**Table 2:** Calculated Radiative Relaxation Mean Lifetimes for the  (τ_r,b_) and the  (τ_r,a_) emissions as well as for the emission of the first excited state L_a_, L_b_ equilibrium mixture τ_r_ = (*f*_b_/τ_r,b_ + *f*_a_/τ_r,a_)^−1^^a^

τ_r,b_ (ns)	τ_r,a_ (ns)	τ_r_ (ns)	τ_r,exp_ (ns)
56	20	22	16.9,^48^ 14.29^49^
			19.3,^50^ 19.9^51^

a The unreported noise is below 0.1 ns. In the table, we also show for comparison the experimental radiative mean lifetime τ_r,exp_ as reported in the available literature.

**Table 3:** Free Energy Change Δ*A* for the L_b_ → L_a_ Transition, the Equilibrium Constant *K*_eq_ = *f*_a_/*f*_b_, and the L_b_ and L_a_ State Equilibrium Fractions *f*_b_ and *f*_a_^a^

	Δ*A* (kJ mol^–1^)	*K*_eq_	*f*_b_	*f*_a_
L_b_ ⇌ L_a_	–5.01 ± 0.50	7.44 ± 1.49	0.118 ± 0.025	0.882 ± 0.025

a (with  the L_b_ → L_a_ transition energy). The noise shown corresponds to a standard error.

The resulting relaxation properties are shown in Tables 2 and 4 where the calculated radiative τ_r_, the nonradiative τ_nr_ mean lifetimes and the fluorescence mean lifetime τ_F_ = (1/τ_r_ + 1/τ_nr_)^−1^ are compared to the corresponding experimental values. From these last two tables, it is evident that our theoretical–computational approach rather well reproduces the experimental relaxation rate constants as obtained by the fluorescence signal (providing quantum yield values of 0.24–0.27 versus our calculated value of 0.15), thus showing that the model employed captures the essential features of the aqueous indole excited state relaxation mechanism. It is worth noting that in our theoretical–computational model we disregarded any L_a_ → triplet and L_b_ → triplet transition, possibly contributing to the nonradiative relaxation involved in the singlet first (perturbed) excited state fluorescence time-dependent signal. However, such intersystem crossing (ISC) processes are characterized by very slow kinetics due to the tiny diabatic state coupling of the ISC reactions (i.e., the spin–orbit coupling^17^), typically resulting in a tiny adiabatic fraction. Therefore, given the high computational costs for including all the possible L_b_ and L_a_ to triplet state transitions and relying on their relevantly slower kinetics compared to the L_a_ → πσ* and L_b_ → πσ* reactions, in our model we did not include the ISC transitions as they can be considered negligible for reconstructing the essential features of the relaxation mechanism. Indeed the only experimental (indirect) estimate of the overall ISC rate constant involved in the aqueous indole singlet first perturbed excited state relaxation we found in the literature^48^ (*k*_ISC_ = 7.6 × 10^–2^ ns^–1^, τ_ISC_ = 1/*k*_ISC_ = 13.2 ns), when included in our calculations provides τ_nr_ = 3.1 ns and τ_F_ = 2.7 ns deviating for only a couple of standard errors from the corresponding values as obtained neglecting the ISC reactions (see Table 4): i.e., the ISC effects are within the statistical noise. Moreover, the kinetic model used to obtain *k*_ISC_ from the experimental data^48^ (the Stern–Volmer quenching model) based on assuming a fast relaxation of the chromophore–quencher complex compared to the chromophore–quencher association and dissociation rates might be inaccurate to treat the slow ISC relaxation, thus possibly resulting in an overestimated *k*_ISC_ (according to preliminary calculations based on our general model for ISC reactions^17^ it is likely that for the first excited state relaxation of aqueous indole τ_ISC_ > 20 ns).

**Table 4:** Calculated Nonradiative Relaxation τ_nr_ = (*f*_b_/τ_b_ + *f*_a_/τ_a_)^−1^ and Fluorescence τ_F_ = (1/τ_r_ + 1/τ_nr_)^−1^ Mean Lifetimes of the First Excited State L_a_, L_b_ Equilibrium Mixture^a^

τ_nr_ (ns)	τ_F_ (ns)	τ_nr,exp_ (ns)	τ_F,exp_ (ns)
4.0 ± 0.4	3.4 ± 0.3	5.9,^48^ 5.5^49^	4.4,^48^ 4.0^49^
		6.4,^50^ 6.3^51^	4.8,^50^ 4.8^51^

a τ_b_ and τ_a_ are the reaction mean lifetimes for the L_b_ → πσ* and L_a_ → πσ* transitions. The noise shown corresponds to a standard error. For comparison, the experimental mean lifetimes τ_nr,exp_ and τ_F,exp_ available in the literature are reported.

## Conclusions

In this paper, we investigated in detail the relaxation process of aqueous indole first excited electronic state. By means of a theoretical–computational model, we reconstructed all the relevant paths involved in the relaxation kinetics, evaluating all the corresponding rate constants. Comparison with all the available experimental data (as provided by the time-dependent fluorescence signal) shows that the model employed rather well reproduces both the radiative and nonradiative mean lifetimes, demonstrating its reliability in elucidating the essential features of the relaxation mechanism. Our results indicate that the relaxation process involves a very fast L_b_*⇌* L_a_ interconversion followed by the much slower L_b_ and L_a_ radiative relaxations competing with the (nonradiative) virtually irreversible transitions to the dark state (i.e., L_b_ → πσ* and L_a_ → πσ*, see Figure 6), then resulting in a relaxation process always involving the L_b_, L_a_ equilibrium mixture. Such L_b_*⇌* L_a_ equilibrium mixture, with a ≈90% L_a_ population, as well as the about 3-fold faster L_a_ nonradiative decay compared to the L_b_ one, make the singlet first (perturbed) excited state relaxation largely characterized by the L_a_ radiative and nonradiative competing decays. It is worth noting that the final relaxation from the dark state to the ground state, being a nonradiative process (i.e., the corresponding radiative relaxation is fully negligible), cannot be detected by fluorescence experiments and thus disregarded in our calculations. Finally, the results obtained, confirming the accuracy of the theoretical approach used for modeling the electronic state transitions, show that the use of a coherent theoretical–computational model can provide a quantitative description of a complex molecular process, unveiling its still controversial and not fully clarified essential features.

## Supporting Information Available

Additional data on the gas phase electronic properties of indole and on the error estimation of the simulated reaction mean lifetimes (PDF)
