# Supplementary material for: Unveiling the Excited State Dynamics of Indole in Solution
Source: J Chem Theory Comput. 2023 Jun 17;19(13):4114–24. doi: 10.1021/acs.jctc.3c00221 (PMC10339669; doi:10.1021/acs.jctc.3c00221)
Supplement: Supplementary file 1 — ct3c00221_si_001.pdf [file ct3c00221_si_001.pdf]

Supporting Information: Unveiling the excited state dynamics of Indole in solution

Cheng Giuseppe Chen,[†] Mauro Giustini,[†] Marco D'Abramo,[*,†] and Andrea Amadei[*,‡]

†*Department of Chemistry, Sapienza University of Rome, Rome 00185, Italy*

‡*Department of Technological and Chemical Sciences, Tor Vergata University of Rome, Rome 00133, Italy*

E-mail: marco.dabramo@uniroma1.it; andrea.amadei@uniroma2.it

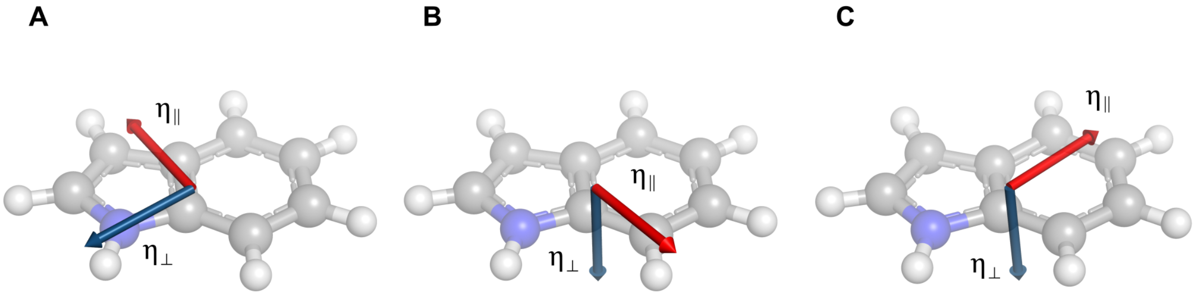

Figure S1: $\boldsymbol{\eta}_{\parallel}$ (red arrow) and $\boldsymbol{\eta}_{\perp}$ (blue arrow) for the $\mathrm{L_b} \longrightarrow \mathrm{L_a}$ (A), $\mathrm{L_b} \longrightarrow \pi\sigma^*$ (B) and $\mathrm{L_a} \longrightarrow \pi\sigma^*$ (C) transitions.

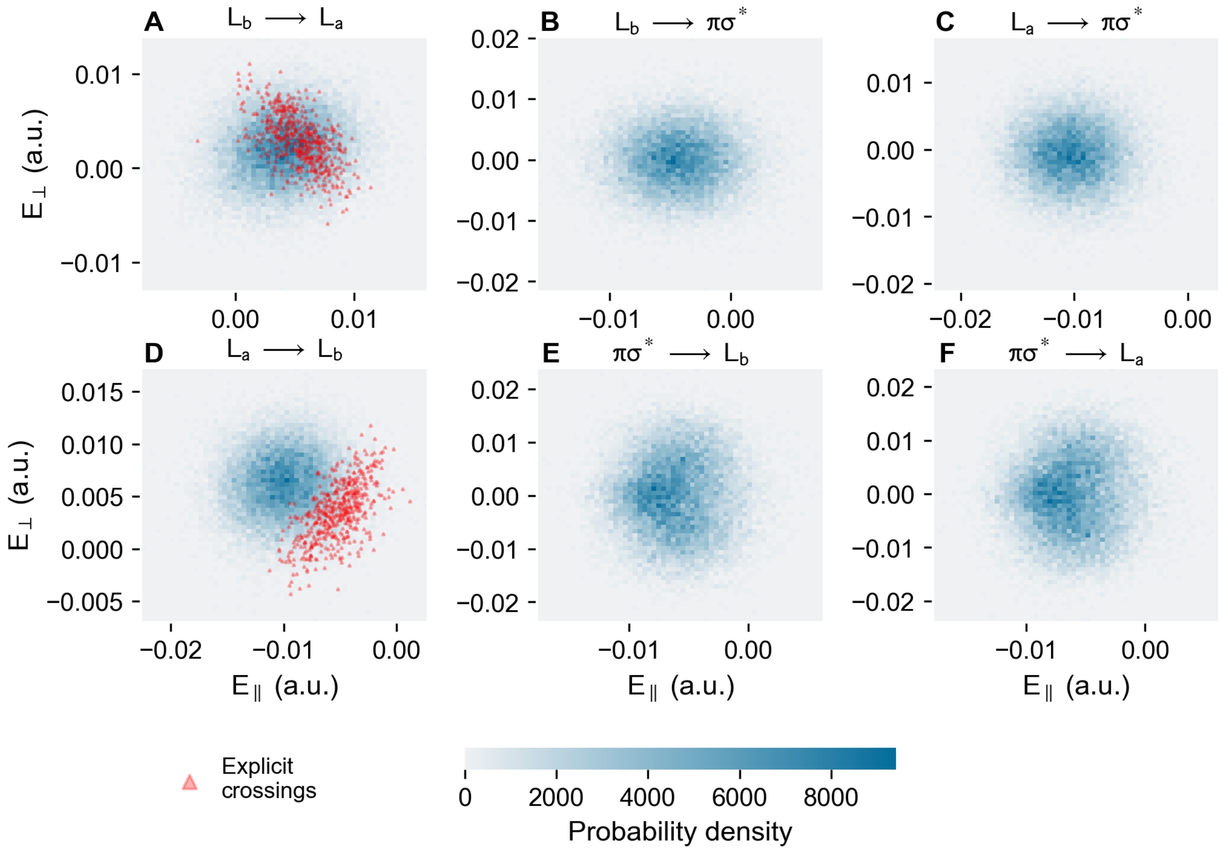

Figure S2: Distributions of the pair values of the projection of the perturbing electric field onto $\boldsymbol{\eta}_{\parallel}$ ($E_{\parallel}$) and $\boldsymbol{\eta}_{\perp}$ ($E_{\perp}$) sampled in the equilibrium MD simulations (blue). Values for the following processed: (A) $\mathrm{L_b} \longrightarrow \mathrm{L_a}$ transition within the $\mathrm{L_b}$ ensemble. (B) $\mathrm{L_b} \longrightarrow \pi\sigma^*$ transition within the $\mathrm{L_b}$ ensemble. (C) $\mathrm{L_a} \longrightarrow \pi\sigma^*$ transition within the $\mathrm{L_a}$ ensemble. (D) $\pi\sigma^* \longrightarrow \mathrm{L_b}$ transition within the $\mathrm{L_a}$ ensemble. (E) $\mathrm{L_a} \longrightarrow \mathrm{L_b}$ transition within the $\pi\sigma^*$ ensemble. (F) $\mathrm{L_a} \longrightarrow \mathrm{L_a}$ transition within the $\pi\sigma^*$ ensemble. The values of $E_{\parallel}$ and $E_{\perp}$ as obtained at the sampled crossings are also shown (the red triangles in panels A and D). It is worth noting that although from the figure it is evident that the equilibrium fluctuations of the two perturbing field components are fully uncorrelated (as required by the crossing mean coupling approximation), when considering only the MD sampled crossings some correlation appears. This discrepancy follows from the use of the atomic charges in the evaluation of the transition energy, resulting in diabatic energy crossings corresponding to a set of correlated $E_{\parallel}$ and $E_{\perp}$ values when the perturbing field is not exactly homogeneous over the QC size. When assuming a fully homogeneous perturbing field and thus the simpler dipolar approach can be utilized to obtain the transition energy, the diabatic energy crossings are all obtained at a fixed $E_{\parallel}$ value with $E_{\perp}$ freely fluctuating. Such an assumption/approximation is what we use within the crossing mean coupling approximation in order to obtain the transmission coefficient to be used when no explicit crossing sampling is available.

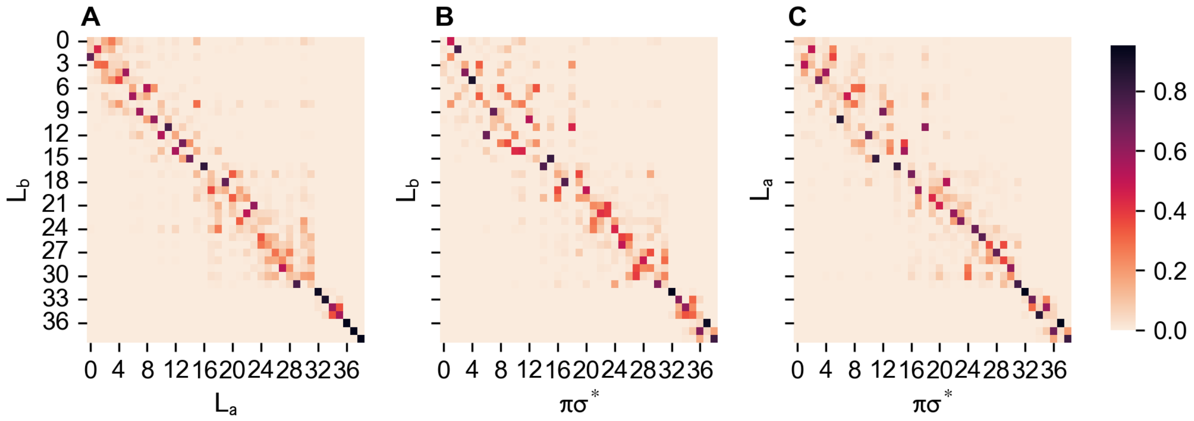

Figure S3: Matrices representing the squared elements of the Duschinsky matrix related to the $L_b \longrightarrow L_a$ (A), $L_b \longrightarrow \pi\sigma^*$ (B) and $L_a \longrightarrow \pi\sigma^*$ (C) transitions.

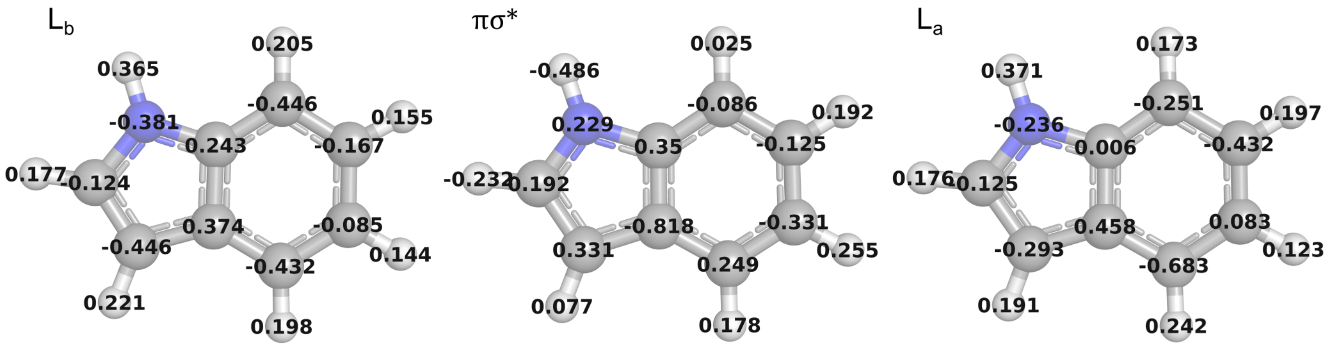

Figure S4: RESP charges obtained for the considered excited electronic states of indole (L_b, $\pi\sigma^*$ and L_a) at the EOM-CCSD/6-311+G(d) using the structures corresponding to the energy minimum of each state.

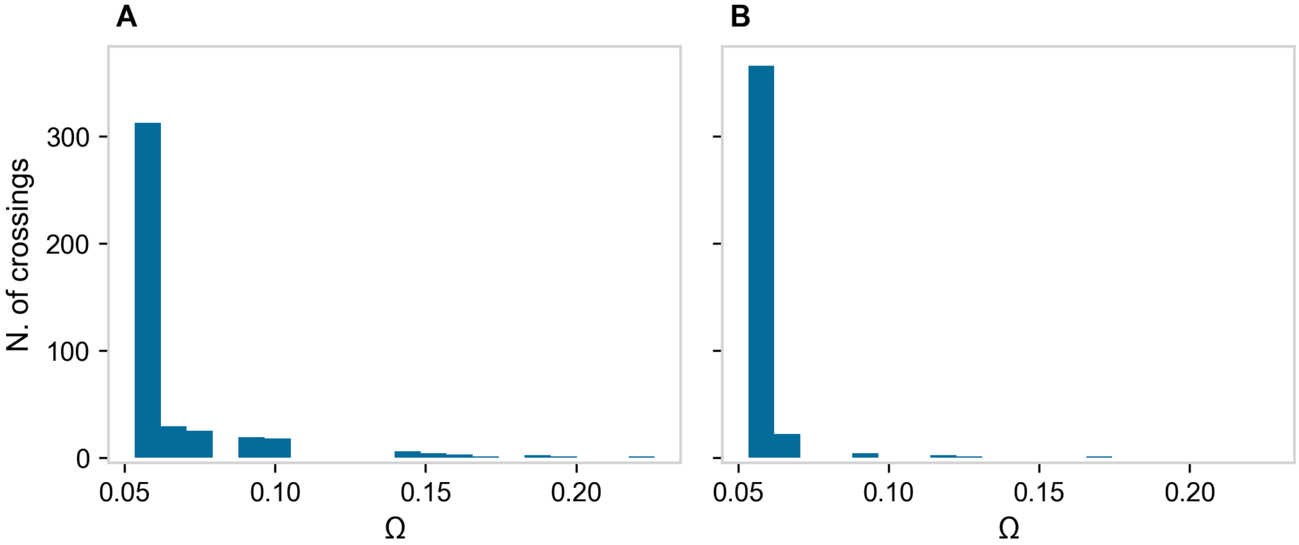

Figure S5: Distributions of the values of Ω sampled for the $L_b \longrightarrow L_a$ transition in the L_b ensemble (A) and $L_a \longrightarrow L_b$ transition in the L_a ensemble (B).

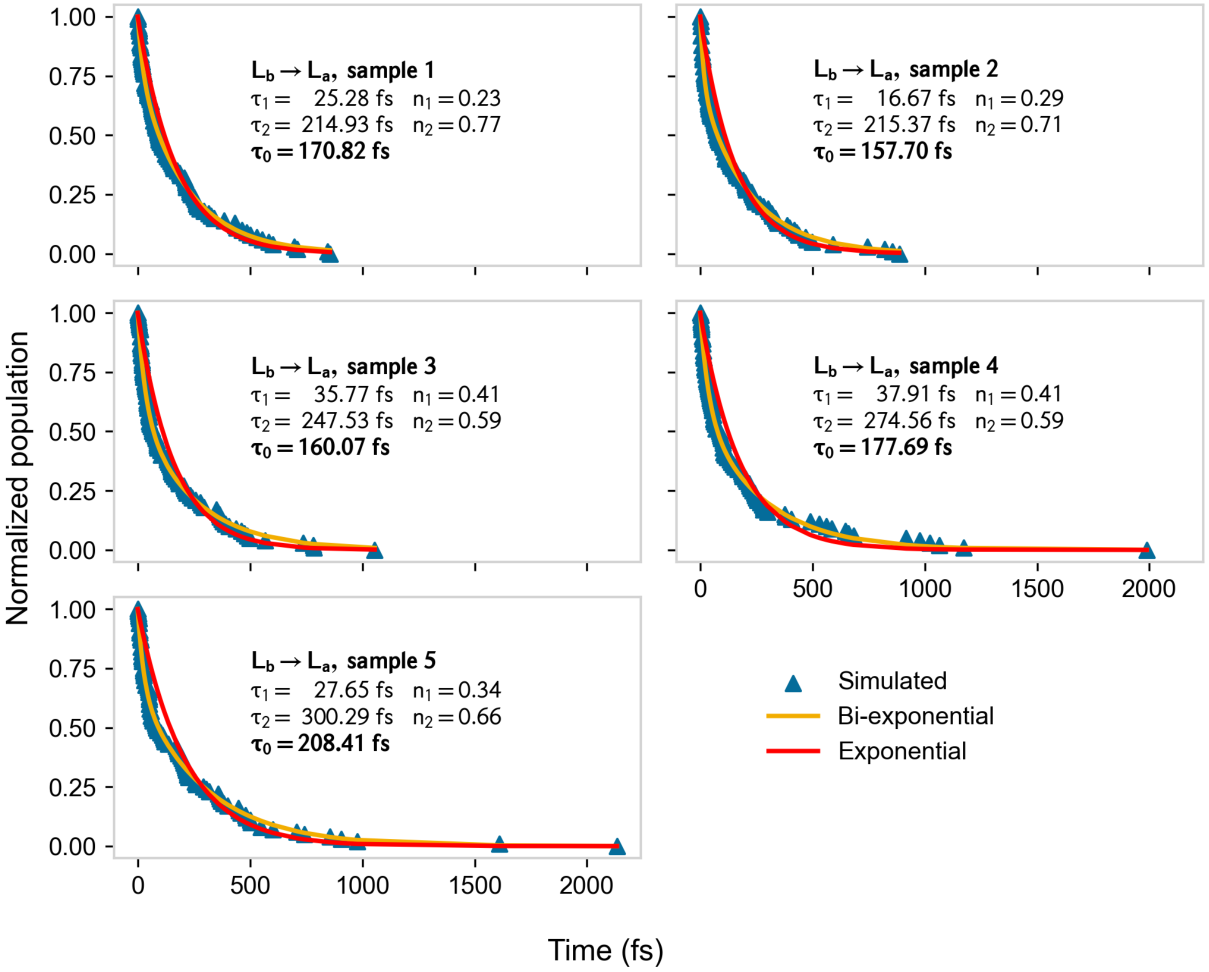

Figure S6: Kinetic traces (blue triangles) associated to the fully adiabatic $L_b \longrightarrow L_a$ transition as obtained monitoring the corresponding diabatic energy crossings provided by 5 samples, each of 100 different MD trajectories. The fitted bi-exponential curves $n_1 e^{-t/\tau_1} + n_2 e^{-t/\tau_2}$ with $n_1 + n_2 = 1$ (yellow lines) are shown, as well as the exponential curves e^{-t/τ_0} (red lines) each resulting from the mean lifetime τ_0 as obtained by the weighted average of the corresponding fitted bi-exponential curve mean lifetimes (i.e., $\tau_0 = n_1\tau_1 + n_2\tau_2$).

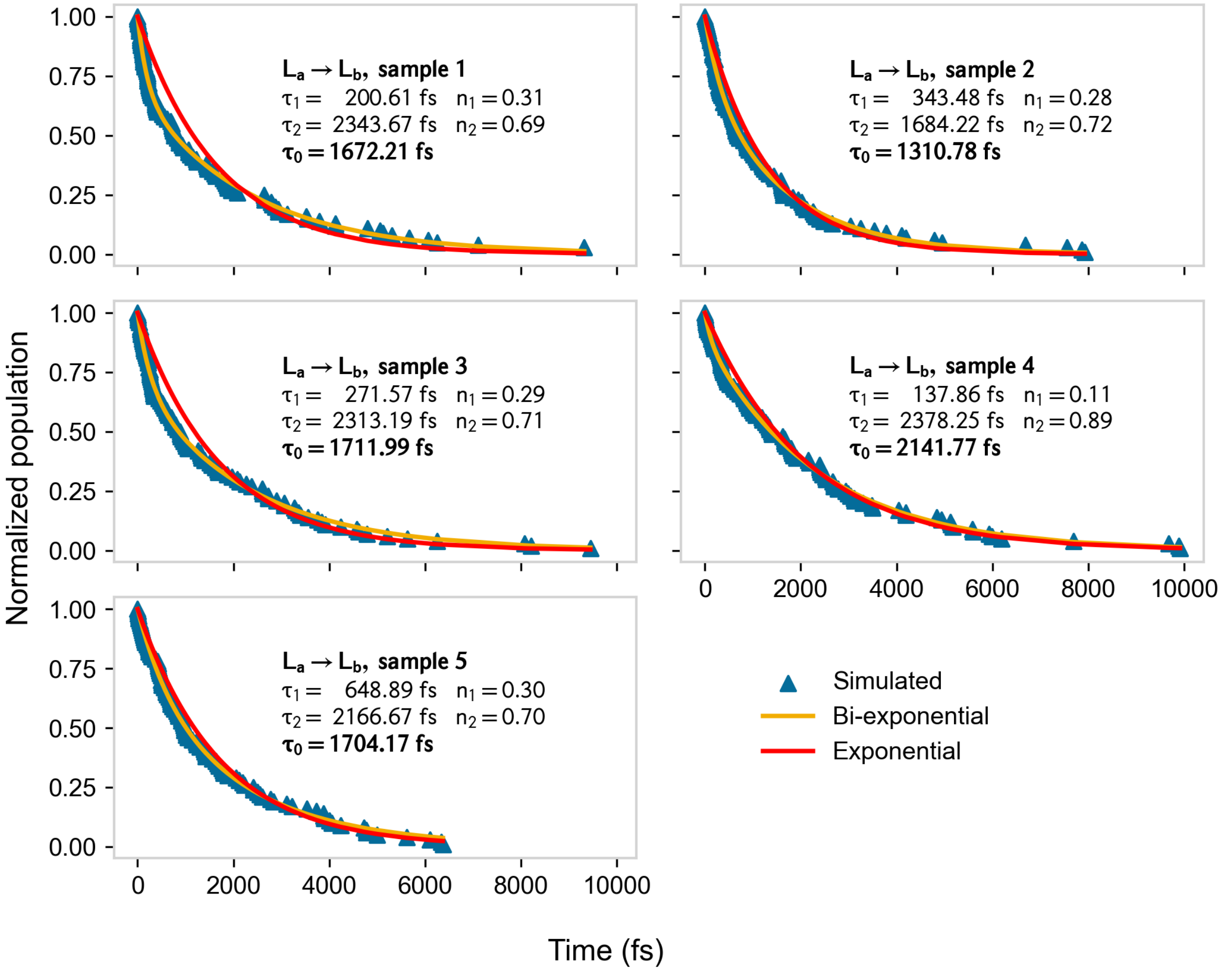

Figure S7: Kinetic traces (blue triangles) associated to the fully adiabatic $L_a \longrightarrow L_b$ transition as obtained monitoring the corresponding diabatic energy crossings provided by 5 samples, each of 100 different MD trajectories. The fitted bi-exponential curves $n_1 e^{-t/\tau_1} + n_2 e^{-t/\tau_2}$ with $n_1 + n_2 = 1$ (yellow lines) are shown, as well as the exponential curves e^{-t/τ_0} (red lines) each resulting from the mean lifetime τ_0 as obtained by the weighted average of the corresponding fitted bi-exponential curve mean lifetimes (i.e., $\tau_0 = n_1\tau_1 + n_2\tau_2$).
